# DNA Condensation by Partially Acetylated Poly(amido amine) Dendrimers: Effects of Dendrimer Charge Density on Complex Formation

**DOI:** 10.3390/molecules180910707

**Published:** 2013-09-03

**Authors:** Shi Yu, Ming-Hsin Li, Seok Ki Choi, James R. Baker, Ronald G. Larson

**Affiliations:** 1 Department of Chemical Engineering, University of Michigan, Ann Arbor, MI 48109, USA; 2 Michigan Nanotechnology Institute for Medicine and Biological Sciences, University of Michigan, Ann Arbor, MI 48109, USA; 3 Department of Internal Medicine, University of Michigan, Ann Arbor, MI 48109, USA

**Keywords:** DNA condensation, dendrimer acetylation, cooperative binding

## Abstract

The ability of poly(amido amine) (or PAMAM) dendrimers to condense semiflexible dsDNA and penetrate cell membranes gives them great potential in gene therapy and drug delivery but their high positive surface charge makes them cytotoxic. Here, we describe the effects of partial neutralization by acetylation on DNA condensation using light scattering, circular dichroism, and single molecule imaging of dendrimer-DNA complexes combed onto surfaces and tethered to those surfaces under flow. We find that DNA can be condensed by generation-five (G5) dendrimers even when the surface charges are more than 65% neutralized, but that such dendrimers bind negligibly when an end-tethered DNA is stretched in flow. We also find that when fully charged dendrimers are introduced by flow to end-tethered DNA, all DNA molecules become equally highly coated with dendrimers at a rate that becomes very fast at high dendrimer concentration, and that dendrimers remain bound during subsequent flow of dendrimer-free buffer. These results suggest that the presence of dendrimer-free DNA coexisting with dendrimer-bound DNA after bulk mixing of the two in solution may result from diffusion-limited irreversible dendrimer-DNA binding, rather than, or in addition to, the previously proposed cooperative binding mechanism of dendrimers to DNA.

## 1. Introduction

Macroions such as cationic dendrimers, polylysine, poly(ethylene imine), and surfactants can be used to condense semiflexible DNA, and so serve as potential substitutes for viral vectors in gene delivery [1,2,3,4,5]. A large number of studies on DNA condensation by poly(amidoamine) or PAMAM dendrimers have been performed because of its great potential as a gene carrier. The interaction between PAMAM dendrimer and DNA is mainly electrostatic, as is the case with histones and DNA, and dendrimers of generation 5 or 6 have diameters roughly comparable to those of histones. Therefore, PAMAM dendrimers also provide an interesting model to study some facets of chromatin formation. To determine how the interaction between dendrimers and DNA affects complex formation, the dendrimer size or generation [6], salt concentration [7], pH [8], and surface groups [9] have been varied, and the morphologies and local structures of the resulting DNA-dendrimer complexes have been resolved using cryo-TEM [10] and small angle X-ray scattering [8]. The binding between fully charged dendrimers and DNA has been shown to be irreversible using optical tweezers to pull on dendrimer-bound DNA without dislodging the bound dendrimers [11].

Although PAMAM dendrimers can bend and condense semiflexible DNA, fully charged dendrimers are toxic to living cells because of their highly charged surfaces. Thus, reducing dendrimer surface charge density by PEGylation [12] or acetylation can be very important for the application of dendrimers *in vivo*. Besides, varying the surface charge density of nanoparticles might be used to improve understanding of chromatin formation, since dendrimers with the same size and charge density as histones can be used to compact DNA and explore whether some aspects of chromatin formation can thereby be induced. Various models of polyelectrolyte condensation by macroions have [13,14] been proposed, which can also be validated if the surface charge density of the dendrimer can be accurately controlled. In addition, weakly charged nanoparticles may melt rather than bend dsDNA [15], which is also of great interest.

In this work, we systematically investigate the interactions between DNA and dendrimers acetylated to various extents using dynamic light scattering, fluorescence spectroscopy, circular dichroism, and fluorescence microscopy imaging. The aim is to help find the appropriate charge density of PAMAM dendrimer for condensing DNA with less cytotoxicity and to help test and clarify the proposed cooperative binding mechanism [1,16] of the DNA-dendrimer interaction.

## 2. Results and Discussion

### 2.1. Dynamic Light Scattering

**Figure 1:**
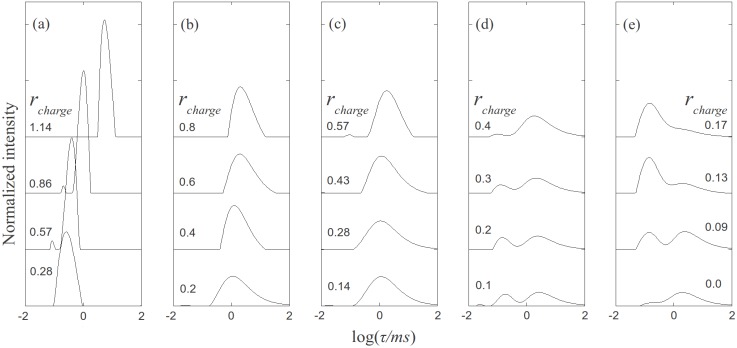
Relaxation time distributions of PAMAM dendrimer/DNA samples measured at scattering angle θ = 50°. (**a**) non-acetylated G5/DNA. (**b**) 30% acetylated G5/DNA. (**c**) 50% acetylated G5/DNA. (**d**) 65% acetylated G5/DNA. (**e**) 85% acetylated G5/DNA.

**Figure 2:**
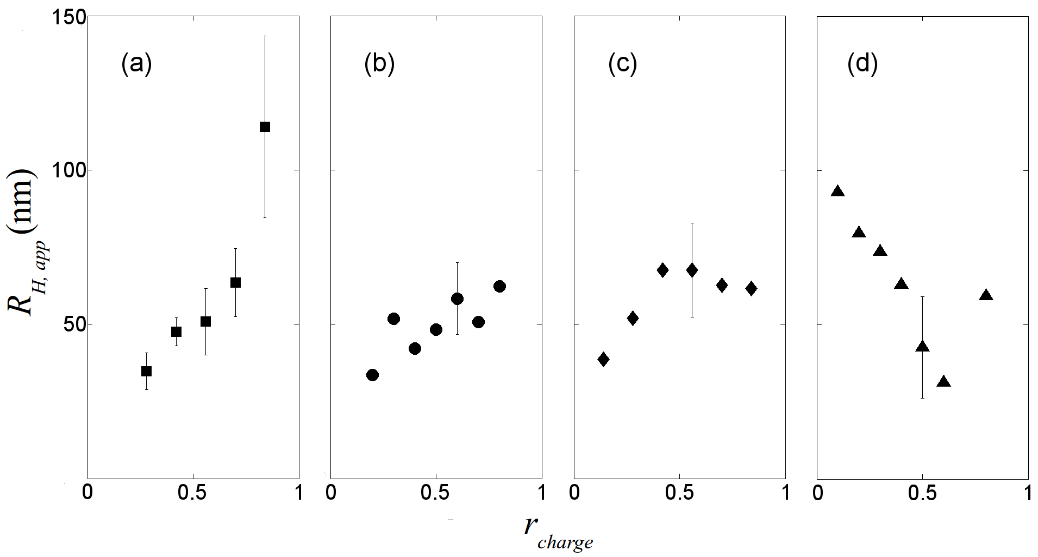
Apparent hydrodynamic radii of dendrimer/DNA complexes measured at θ = 50°. (**a**) non-acetylated G5/DNA. (**b**) 30% acetylated G5/DNA. (**c**) 50% acetylated G5/DNA. (**d**) 65% acetylated G5/DNA. Some typical error bars are given.

DNA and PAMAM dendrimers (generation 5) were acetylated to various extents, mixed in 10 mM NaBr solutions, and studied by dynamic light scattering. According to our titration experiments (data not shown here), the number of charges on non-acetylated G5 dendrimers is 114, instead of 128, the number of positive charges on a perfectly synthesized dendrimer [17]. Therefore, assuming that defects that lead to the reduced charge occur randomly, the standard deviation of number of charges on a non-acetylated dendrimer can be approximated by: 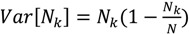, where N is the maximum number of charges possible, which equals 128, and N_k_ is the measured mean value of 114. If the acetylation of dendrimer terminal groups is also taken to be random, the standard deviation of the number of charges on an individual acetylated dendrimer can also be approximated by the equation above. Thus, the numbers of positive charges, and their standard deviations computed by propagation of errors using both sources of variation mentioned above, on dendrimers acetylated to 0%, 15%, 30%, 50%, 65%, and 85%, are 114 ± 3.5, 97 ± 5.2, 80 ± 6.0, 57 ± 6.5, 40 ± 6.2, 17 ± 4.0, respectively. The relaxation time distributions of pure dsDNA as well as DNA-dendrimer complexes were obtained from autocorrelation functions of scattering light intensities using analysis with CONTIN 2DP. Selected relaxation time distributions of DNA-dendrimer complexes are shown in Figure 1 with scattering angle θ fixed at 50°. The apparent hydrodynamic radii of the DNA-dendrimer complexes were computed using the peaks of the relaxation time distributions along with the Einstein-Stokes equation, and plotted in Figure 2. Note that *r_charge_* here and in the following discussion is defined as the ratio of the total number of positive charges on all dendrimers to the total number of negative charges on all DNA molecules in the solution, that is, *r_charge_* = [NH_3_^+^]/[PO_4_^−^]. All the primary amine groups (pK_a_ = 9.0 [18] ~10.77 [19]) were assumed to be protonated in pH 7~8 in this study. This assumption was supported by titration experiments and the observation that solutions become cloudy when *r_charge_* defined above is close to unity. In this paper, we limit ourselves to the cases with *r_charge_* less than 1 to study DNA-dendrimer complexes before phase separation takes place. 

The relaxation time distribution of 0.15 mg/mL pure salmon sperm DNA (2000 ± 500 bp) in 10 mM NaBr solution is plotted in Figure 1e, where *r_charge_* is 0. The two peaks of the DNA relaxation time distribution correspond to the internal (shorter τ) and translational (longer τ) modes of DNA. The apparent hydrodynamic radius of DNA computed based on the translational peak is 110 nm, in agreement with previous studies [1,6]. DNA concentrations for all dynamic light scattering measurements were fixed at 0.15 mg/mL, while the dendrimer concentration was varied to obtain the specified r_charge_. The apparent hydrodynamic radius of the G5 PAMAM dendrimer was determined to be around 3 nm by DLS, indicating that aggregation of dendrimer in the absence of DNA was negligible.

As shown in Figure 1a, non-acetylated G5 dendrimer-DNA complexes give sharp peaks, indicating that the complexes size distribution is relatively narrow. Comparing the positions of these peaks to those of the pure DNA, we can conclude that the former, which correspond to faster relaxation, and therefore smaller objects, represent condensed DNA-dendrimer complexes. It should be noted that values of *r_charge_* of 0.28, 0.57, 0.86, and 1.14 correspond to *r_molar_* values of 10, 20, 30, and 40, where *r_molar_* is defined by [dendrimer]/[DNA]. The decay time distributions of the acetylated denrimer-DNA complexes with the same molar ratios (*r_molar_*) are plotted in Figure 1b–e, except for the pure DNA curve at the bottom of Figure 1e. For the 30% acetylated dendrimer (Figure 1b) and 50% acetylated dendrimer (Figure 1c), the decay time distributions are much broader than for the non-acetylated dendrimer in Figure 1a, probably because the number of positive charges on the acetylated dendrimers varies somewhat from dendrimer to dendrimer, as estimated in the standard deviations in numbers of charges given above. For the dendrimers with the highest acetylation ratios (65%, 85%), the relaxation time distributions exhibit two peaks, the shorter of which is presumably contributed by internal motion within the DNA complex, while the slower mode corresponds to translational motion of the complexes. However, the slower mode for 85% acetylated dendrimer-DNA is almost the same as for pure DNA, indicating that the weakly charged dendrimer (~15 charges per dendrimer) was not able to condense the DNA significantly. This result agrees with a previous study of DNA/Poly-L-lysine [3], which showed that a transition of the DNA-polycation complex conformation occurs when the number of charges on the polycation is decreased. Highly charged polycations can bend and condense dsDNA while weakly charged polycation only attach to it without producing compaction.

The relaxation time distributions of acetylated dendrimer-DNA complexes with higher molar ratios (above 40) are not presented here, since for these cases a small fraction of the most highly charged dendrimers from the polydisperse charge distribution can condense the DNA even when the average charge per dendrimer is low and this small fraction dominates the dynamic light scattering signals.

The apparent hydrodynamic radii of the DNA-dendrimer complexes are plotted in Figure 2. For highly charged dendrimers (0%, 30%, 50% acetylated), the hydrodynamic radii of the complexes are around 50 nm. Therefore, this level of acetylation of primary amine groups on the PAMAM dendrimer does not significantly reduce its ability to condense DNA, despite the lessening of the dendrimer charge, which is needed for condensation [14]. Since reduction of the charge density on the dendrimer can reduce the cytotoxicity of dendrimer significantly [12], partial acetylation of the primary amine groups of PAMAM dendrimer might have significant potential in gene therapy. For highly charged dendrimers, as *r_charge_* increases, the apparent hydrodynamic radius of the complexes also increases (Figure 2a), which is likely the result of formation of large complexes containing more than one DNA chain. However, for weakly charged dendrimers (65% acetylated), the complex radius decreases when *r_charge_* is increased up to 0.5, as the weakly charged dendrimer at low concentration binds to DNA without compaction, but compaction apparently increases with increasing numbers of bound dendrimers per DNA molecule. Based on our dynamic light scattering experiments, to condense DNA in the gene delivery process, PAMAM dendrimers with around half of their primary amine groups acetylated might be a good choice. To determine whether the polydispersity of charges on acetylated dendrimer will affect the gene delivery, transcription experiments should be performed.

### 2.2. Steady-State Fluorescence Spectroscopy

**Figure 3:**
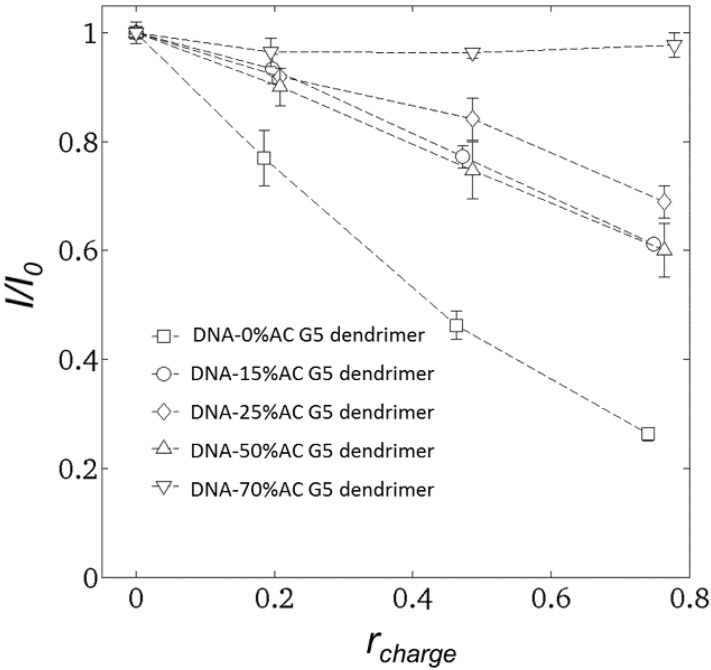
Emission light intensity (527 nm) versus *r_charge_* for DNA condensed by dendrimers with various acetylation ratios. DNA-0% acetylated dendrimer (squares); DNA-15% acetylated dendrimer (circles); DNA-25% acetylated dendrimer (diamonds); DNA-50% acetylated dendrimer (up triangles); DNA-70% acetylated dendrimer (down triangles).

To determine the fraction of free DNA in the DNA-dendrimer mixture, steady-state fluorescence spectroscopy experiments were carried out 10 min after mixing DNA-dendrimer complex solutions with nucleic acid stain GelStar^®^. The excitation wavelength was fixed at 493 nm, and emission light intensity was recorded at 527 nm. The normalized emission light intensity of dendrimer-DNA complexes for various dendrimer concentrations are plotted in Figure 3.

GelStar^®^ was assumed to bind only to free DNA, that is to portions of the DNA not blocked by dendrimer [1]. After reacting GelStar^®^ with DNA, the emission light intensity increased dramatically although the emission from free GelStar^®^ was negligible. Previous studies [1,6] assumed that GelStar^®^ is not able to react with dendrimer-bound DNA. This is validated by our non-acetylated G5-dendrimer results, for which the normalized emission light intensity is roughly equal to (1-*r_charge_*); see Figure 3. Therefore, GelStar^®^ only binds to the free DNA segments which have not been neutralized by dendrimer, since the interaction between DNA and GelStar^®^ is also dominated by electrostatic force. However, the relation *I/I_0_* = (1 − *r_charge_*) does not hold for DNA-acetylated dendrimers. This is apparently because the binding affinity of acetylated dendrimers to DNA decreases as the acetylation ratio increases. One implication is that the DNA segments condensed by dendrimer with fewer charges might still be accessible to proteins.

### 2.3. Circular Dichroism Spectroscopy

**Figure 4:**
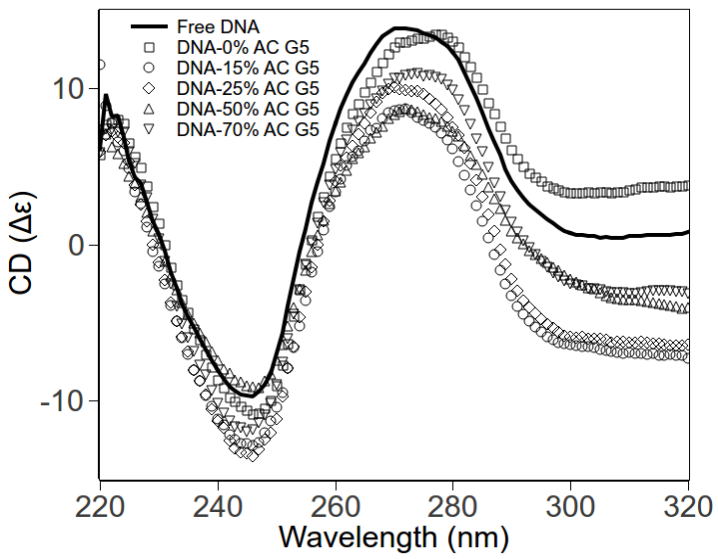
CD spectra of pure DNA and DNA-PAMAM dendrimer complexes in HEPES (pH 7.3). Free DNA (solid line); DNA-0% acetylated dendrimer (squares); DNA-15% acetylated dendrimer (circles); DNA-25% acetylated dendrimer (diamonds); DNA-50% acetylated dendrimer (up triangles); DNA-70% acetylated dendrimer (down triangles).

Using circular dichroism (CD) spectroscopy, we explore the DNA conformations within DNA-dendrimer complexes. The DNA concentrations were fixed, with *r_charge_* also fixed to 0.2 for various dendrimers. The CD spectra for different complexes are shown in Figure 4. The negative peak (245 nm) and positive peak (275 nm) of free DNA are consistent with CD spectra of B-form dsDNA [20,21]. CD spectra of DNA-dendrimer complexes exhibit the same shape as the free B-form dsDNA. The shift of peaks is negligible. Therefore, at low charge ratio (*r_charge_*), the DNA in DNA-acetylated dendrimer complexes remains in the classical B-form. In other words, dendrimers and acetylated dendrimers bend DNA without disrupting the local helical structure of DNA.

### 2.4. Molecular Combing Assay

**Figure 5:**
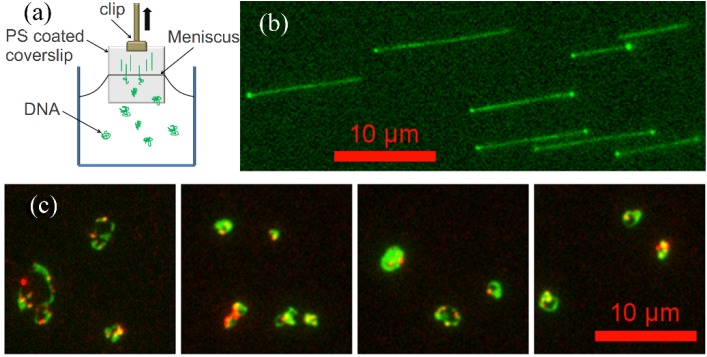
(**a**) Experimental setup for molecular combing. (**b**) Immobilized, aligned, YOYO-1 stained λ-DNA on PS surface. (**c**) λ-DNA/G5 PAMAM dendrimer (TRITC labeled) complexes deposited on PS coated cover glass surface (green: λ-DNA, red: PAMAM dendrimer).

Linearized λ-DNA has a hydrophobic 12-base overhang on each end, which allows it to stick and anchor at each end to a polystyrene-coated cover glass [22,23]. When the PS coated cover glass is pulled out from DNA solution, the YOYO-1 stained free DNA molecules are aligned on the cover glass surface by the high air-water surface tension, and then are visualized by a fluorescence microscope with blue excitation (Figure 5b). We also fluorescently labeled dendrimers with amine-reactive tetramethylrhodamine isothiocyanate (TRITC) dye molecules. TRITC-labeled PAMAM dendrimer-DNA complexes were visualized by sequentially imaging DNA and dendrimer molecules using blue and green excitation, respectively. The overlays of DNA and dendrimer images are shown in Figure 5c. Since the λ-DNA molecules were condensed by dendrimer, even the high surface tension (up to 4.0 × 10^−10^ N) is not able to stretch the DNA out to its full contour length (16 μm). Thus, we are able to image the condensed form of λ-DNA directly. Here, we only present images of dendrimer-DNA complexes at low molecular ratio (100 dendrimers per DNA) where the dendrimer-DNA complexes are somewhat condensed but do not form dense globular particles, and a few complexes stick to the PS surface so that we can image them. For dendrimer-DNA complexes with higher molecular ratio (for example, around 1,000 dendrimer/DNA), the complexes do not stick to the surface, possibly because the two hydrophobic ends of the λ-DNA at these ratios are trapped within the denser complex particle, preventing the DNA from binding to the PS surface. 

In the dendrimer-DNA complexes, as shown in Figure 5c, the dendrimer molecules concentrate along some regions of the DNA, while other regions remain dendrimer free. This is consistent with a previous study [1] in which dendrimer-bound DNA was shown to coexist with dendrimer-free DNA. These earlier results were interpreted as evidence that the dendrimer molecules bind to the DNA in a “cooperative” manner, in which dendrimers have higher binding affinity to DNA with dendrimers already attached to it than to bare DNA. In what follows, we suggest that instead of, or along side of, cooperative binding, that irreversible diffusion-limited binding might help account for these results. In any event, the dendrimer-DNA complexes, once formed, are strong enough to resist unraveling during combing, implying a resistance to a force of around 500 pN per complex [23]. 

We also incubated λ-DNA molecules aligned on the PS surface with a TRTIC-labeled G5 dendrimer solution, expecting dendrimers might slide one dimensionally along the DNA deposited on the surface, since a previous study [24] revealed that electrostatic forces were sufficient to confine the charged nanoparticle in one dimension. However, rather than experiencing 1D diffusion along DNA as some DNA-binding proteins do, G5 dendrimers either stick to the DNA or the PS-coated surface or performed simple 3D Brownian diffusion (data not shown). The failure to undergo 1D diffusion along DNA might be due to the high binding energy [14] of the irreversible interaction of charged dendrimers with DNA [11].

### 2.5. Flow Stretching Assay

After biotinylated λ-DNA with one end attached to a neutravidin monolayer was deposited on the flow channel surface, the YOYO-labeled DNA was stretched by a shear flow. Based on parabolic flow assumption, at the flow rate imposed, the shear rate at the free end of the DNA, which based on the DNA coil size is estimated to be about 1 µm above the cover glass surface, is around 300 s^−1^ , which is able to stretch the DNA almost to its full contour length.

A TRITC-labeled G5 dendrimer (non-acetylated) solution was then introduced into the flow cell to interact with the tethered λ-DNA. The binding process was visualized and recorded by fluorescence microscopy using green excitation. We observed that the dendrimer bound and decorated the entire λ-DNA molecule, as opposed to the partial binding we observed in the molecular combing assay. When the λ-DNA is fully stretched out, each segment of the DNA apparently has almost the same probability of binding a dendrimer molecule. Apparently because the binding is irreversible and the well-mixed dendrimer solution is continuously injected into the flow channel, the dendrimers can cover the whole DNA chain evenly. After formation of the dendrimer-DNA complex, we used TE buffer to wash out the flow channel. No significant fluorescence decrease was observed on the tethered DNA until photocleavage or neutravidin detachment occurred, usually around 5~10 min after illumination, which confirmed that dendrimer attached to DNA irreversibly.

**Figure 6:**
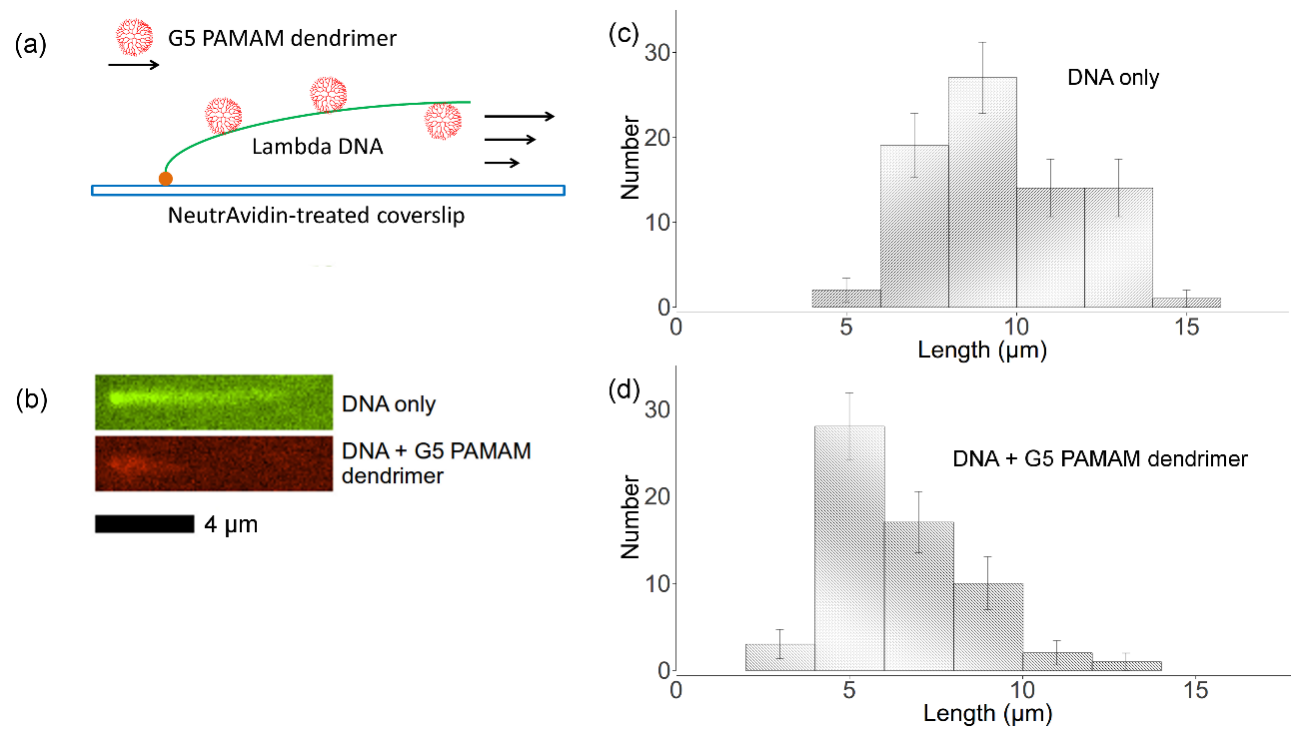
(**a**) Experimental setup for imaging dendrimer binding to flow-stretched DNA with one end tethered to the surface. (**b**) Green: tethered YOYO-labeled λ-DNA in flow; red: tethered TRITC-labeled dendrimer-DNA complex in flow. (**c**) Distribution of free λ-DNA molecules lengths. (**d**) Distribution of dendrimer-DNA complex lengths.

Figure 6c,d show the length distributions of both free DNA and dendrimer-DNA complexes. 77 λ-DNA lengths as well as 62 dendrimer-DNA complexes lengths were recorded for these distributions. The error bar was approximated using equation: 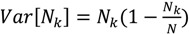, where *N_k_* is the number of data points of *k_th_* bin and *N* is the total number of data points. The lengths distributions are wide, which is mainly due to the photocleavage of λ-DNA, which shortens some of the DNA molecules before imaging is complete. However, these two histograms indicate that flow-stretched tethered DNA molecules are condensed by G5 dendrimer bound to the DNA chains, agreeing with optical tweezer experiments [11].

The interaction between flow-stretched λ-DNA and TRITC-labeled G5 PAMAM dendrimers with 50% primary amine groups acetylated were studied using the same procedures. Because the neutravidin was physically attached to the cover glass, it could easily detach the surface under high drag force. Thus, we only imaged the DNA in flow for less than 30 min. Within this time, binding of 50% acetylated dendrimer to DNA was not observed. This indicates a significantly reduced binding affinity upon partial acetylation of the dendrimer, in agreement with our dynamic light scattering results as well as fluorescence spectroscopy results discussed above.

### 2.6. Cooperative Binding *vs.* Diffusion Limited Reaction

The differences in DNA-dendrimer complex conformations between the molecular combing assay and the flow stretching assay suggest an explanation to the observed apparent “cooperative binding” of dendrimers to DNA. “Cooperative binding” was proposed based on the coexistence of free DNA and compacted DNA-dendrimer complexes [1,16] formed in bulk solution. However, no coexistence of free DNA with complexes was observed in our flow-stretching assay. It is possible that the “cooperative binding” is much stronger in coiled DNA than in stretched DNA, perhaps because of coupling between DNA bending and dendrimer binding. However, another possibility is that kinetics limitation plays a role. According to our imaging results, this irreversible DNA-dendrimer binding reaction can be very fast. For example, the tethered DNA was covered by dendrimer in just minutes when we used 1 nM dendrimer solution, while it is covered by dendrimer instantly within our temporal resolution, which is a few seconds when using a higher concentration of dendrimer (>20 nM). Thus, the formation of DNA-dendrimer complexes is likely diffusion limited. If so, when *r_charge_* is less than unity, coexistence of free DNA and DNA-dendrimer complexes in bulk experiments might be simply due to the presence of free DNA that had not yet encountered free dendrimer before the free dendrimer was exhausted from the solution. Distinguishing between cooperative and simply irreversible binding would require very slow and careful mixing of dendrimers with DNA, to ensure that dendrimers can access every segment of DNA with equal probability before irreversible reaction occurs. 

## 3. Experimental

### 3.1. DNA Preparation

Salmon sperm DNA (2,000 bp, 10.0 mg/mL in TE buffer) was purchased from Invitrogen (Grand Island, NY, USA) and used without further purification. Before using it, the ratio of absorbance at 260 nm to that at 280 nm was checked by a GeneQuant spectrophotometer, and found to be above 1.8, which is required to ensure that protein contamination was negligible. Lambda phage DNA (48.5 kbp) was purchased from New England Biolabs (Ipswich, MA, USA), and heated at 65 °C for 10 min followed by quick cooling to restore the molecules to linear form. For lambda DNA staining, the intercalating dye YOYO-1 (Molecular Probes, Grand Island, NY, USA) was diluted to 100 nM in Tris-EDTA buffer (pH 8.0) and mixed with lambda DNA at a staining ratio of 1 dye per 20 bp for both combing and for the flow-stretching assay. Stained λ-DNA (3 nM) was ligated to a biotinylated 12mer (5'-agg tcg ccg ccc-biotin, 30 nM, Operon) using T4 DNA ligase (New England Biolabs) for flow stretching.

### 3.2. PAMAM Dendrimer Preparation

Generation 5 PAMAM dendrimer was purchased from Dendritech and purified by dialysis against water as described elsewhere [17,25]. The mixtures of acetic anhydride and PAMAM dendrimer were prepared in anhydrous methanol. The reactions between acetic anhydride and dendrimer were carried out in a glass flask at room temperature for 24 h. The ratio between acetic anhydride and dendrimer was varied to synthesize PAMAM dendrimers with various degrees of acetylation. The standard deviation of the average acetylation ratio was about 5%, based on titration experiments. The reaction products were dialyzed first against PBS buffer (pH 8.0) and then against deionized water over night. The purified acetylated PAMAM dendrimer samples were lyophilized and stored at −20 °C. More details about dendrimer acetylation can be found elsewhere [26]. TRITC labeled PAMAM dendrimer was prepared in methanol and purified by 10 KD MWCO dialysis and ultra-filtration. Purified TRITC labeled dendrimer was stored at −20 °C and kept away from light.

### 3.3. Dynamic Light Scattering

Salmon sperm DNA and PAMAM dendrimer with various degrees of acetylation were prepared in 10 mM NaBr solutions and filtered by 0.2 µm Minisart filters (Sartorius, New York, NY, USA). The mixtures of DNA and dendrimer were prepared by adding 500 µL dendrimer solutions into equal volume of DNA solutions. The concentrations of DNA in all mixtures were fixed at 0.15 mg/mL. Dynamic light scattering measurements were carried out at 25 °C after at least 3 h-reaction. Then the pH of mixtures were determined to be 7~8, where the primary amine groups of the PAMAM dendrimer are protonated. More details about DNA dendrimer complex formation can be found elsewhere [1]. All dynamic light scattering experiments were conducted on an ALV (Langen, Hessen, Germany) compact goniometer system. The wavelength of incident laser light was λ = 488 nm (Innova 70C argon ion laser, Coherent Inc., Santa Clara, CA, USA). Scattered light was collected by dual avalanche photodiode detectors, which were in the transmission mode and then sent to a multi tau correlator (ALV-5000E). The time-averaged normalized intensity autocorrelation function was constructed by cross correlating the signal. The hydrodynamic radius of the complex particle was calculated from the decay times using CONTIN 2DP analysis together with the Stokes-Einstein equation. Light scattering angle was fixed at 50° for all measurements.

### 3.4. Fluorescence Spectroscopy

Steady-state fluorescence emission measurements were carried out on Fluoromax-2 fluorimeter using a 10 × 10 mm quartz cuvette (Starna, Atascadero, CA, USA). The excitation wavelength was set to 493 nm. Emission spectra were recorded between 400 nm and 700 nm in 1 nm increments. Integration time was set to 0.1 s. For each sample, 3 scans were accumulated and averaged. Salmon sperm DNA-dendrimer complexes solutions were incubated with equal amount of GelStar^®^ nucleic acid stain for more than 10 min before measurement, and DNA final concentrations were fixed at 2 µg/mL. 

### 3.5. Circular Dichroism Spectroscopy

All circular dichroism (CD) measurements were conducted on an Aviv model 202 circular dichroism spectrometer at 25 °C using a 10 mm quartz cell. Spectra were recorded between 220 nm and 320 nm with 1 nm increments and 1 s averaging time. The bandwidth was set to 1 nm. Five scans were averaged for each sample. Salmon sperm DNA and PAMAM dendrimer with various degrees of acetylation were incubated together to form complexes in 20 mM HEPES buffer for at least 3 h before CD measurements. DNA concentrations in the final solutions were fixed at 65.0 µg/mL. All CD measurements were baseline corrected by subtracting the blank from the recorded spectra.

### 3.6. Molecular Combing Assay

For molecular combing, YOYO-1 stained λ-DNA was diluted into 2 pM in TE buffer (pH 8.0). λ-DNA was mixed with TRITC labeled PAMAM dendrimer to form complexes. For DNA-dendrimer complexes solutions, the ratio between dendrimer and DNA was set to 100. 100 mg/mL polystyrene (MW 10,000, Sigma, St. Louis, MO, USA) solutions were prepared in toluene and spin-coated onto a cover glass (25 × 25 mm, Corning, No. 1) at 3,000 rpm for 30 s. Then the PS-coated cover glasses were each dipped into either DNA solution or DNA-dendrimer complexes solutions for 3 min and then pulled out at a constant rate (200 μm/s) using a linear stage (Zaber, T-LLS series, Vancouver, BC, Canada), as shown in Figure 5a.

### 3.7. Flow Stretching Assay

Cover glasses (25 × 25 mm, Corning, No. 1) and glass slides (Fisher, Pittsburgh, PA, USA) were cleaned by nitric acid and hydrochloric acid and then rinsed by deionized water. Then the cover glass was attached to the slide using double-sided tape to form the bottom wall of a flow channel (length 20 mm, width 8 mm and height 80 μm). The flow channel was incubated first by 5 mg/mL neutravidin (Invitrogen) for 1 h and then by 2 mg/mL α-casein (Sigma) for 0.5 h. Biotinylated λ-DNA (3 nM) was injected into the flow channel and reacted with the neutravidin attached to the flow channel surface for 5 min. Then the flow channel was washed by TE buffer (pH 8.0) for 1 min to expel the free DNA. 1 nM TRITC labeled PAMAM dendrimer in TE buffer was then introduced to flow channel at a constant rate (10 mL/h) using a syringe pump (KD Scientific 100 series) to stretch tethered λ-DNA for 20 min which allowed the dendrimer to bind to the λ-DNA, which is shown in Figure 6a.

### 3.8. Fluorescence Microscopy

YOYO-1-stained λ-DNA and TRITC-labeled PAMAM dendrimer were visualized by a Nikon TE2000-U inverted fluorescence microscope with a front-illuminated CCD camera (Cascade 512F, Roper Scientific, Tucson, AZ, USA). A dual-band excitation and emission FITC-TRITC filter with X-CITE® 120 series lamp were used to illuminate the specimens. The integration time for each frame was set to 200 ms. The camera exposure time and filter wheel shutter were controlled by MetaMorph 7.0.

## 4. Conclusions

In conclusion, PAMAM dendrimers with a fraction of their primary amine groups acetylated have were used to condense DNA and the complexes were characterized using light scattering, circular dichroism, and optical fluorescence microscopy both after combing complexes formed in bulk solution onto a hydrophobic surface, and during complex formation onto flow-stretched DNA tethered onto a surface. Using dynamic light scattering, we confirmed that unless a high fraction (>70%) of primary groups was neutralized the acetylated dendrimer was able to compact DNA even with reduced charge, and therefore presumably with reduced cytotoxicity. The bound DNA retains its B-form, despite the condensation, and condensed form is strong enough to resist the forces created by molecular combing, which are on the order of 500 pN [23]. We imaged this dendrimer-DNA binding process in solution and in real time for the first time. When DNA tethered to a surface was exposed to dendrimer introduced continuously by flow, we observed all DNA molecules gradually load up with dendrimers, which were not washed out by subsequent dendrimer-free solutions. We did not observe co-existence of dendrimer-loaded DNA with dendrimer-free DNA, and at high concentrations of dendrimer, the DNA became loaded with dendrimers almost instantly. Thus, taken together, our single-molecule experimental results indicate that the coexistence of DNA/dendrimer complexes with free DNA observed in bulk solution [1,16] might not be due to cooperative binding of dendrimer to DNA, but rather to diffusion-limited and irreversible reaction between dendrimer and DNA that can occur when dendrimer and DNA are mixed in the bulk.
